# Consensus statement for perioperative care in total hip replacement and total knee replacement surgery: Enhanced Recovery After Surgery (ERAS®) Society recommendations

**DOI:** 10.1080/17453674.2020.1724674

**Published:** 2020-02-14

**Authors:** Thomas W Wainwright

Further to the publication of our article, in light of recent research (Kumin et al. 2019), and the results of forth coming trials, the wording used in regard to the non recommendation of forced air warming devices is currently too strong. We would therefore like to remove the following sentence and references from “Maintaining normothermia,” (page 10) “However, the use of forced air-warming is not recommended as there is evidence that this is associated with an increased risk of infection (McGovern et al. 2011, Koc et al. 2017)”. We provide an updated below.

## ^Maintaining normothermia^

The National Institute for Clinical Excellence (NICE) recommends the pre-warming of patients and to maintain the active warming of all adults undergoing surgery throughout the intraoperative phase (NICE, 2016). Multiple series suggest that normothermia should be targeted as part of the anesthetic care of hip and knee replacement patients. There are many methods described to conserve body temperature, including pre-warming and humidification of anesthetic gases, warming IV and irrigation fluids and forced air warming blankets and devices. In addition, the ambient temperature should be at least 21 °C while the patient is exposed prior to active warming starting (NICE, 2016).

***Summary and recommendations****—Normal body temperature should be maintained peri- and postoperatively through pre-warming and the active warming of patients intraoperatively*

***Evidence level****—High*

***Recommendation grade****—Strong*
